# Self domestication and the evolution of language

**DOI:** 10.1007/s10539-018-9612-8

**Published:** 2018-03-27

**Authors:** James Thomas, Simon Kirby

**Affiliations:** Centre for Language Evolution, University of Edinburgh, 3 Charles Street, Edinburgh, EH8 9AD UK

**Keywords:** Self-domestication, Cultural evolution, Language evolution, Cultural transmission

## Abstract

We set out an account of how self-domestication plays a crucial role in the evolution of language. In doing so, we focus on the growing body of work that treats language structure as emerging from the process of *cultural transmission*. We argue that a full recognition of the importance of cultural transmission fundamentally changes the kind of *questions* we should be asking regarding the biological basis of language structure. If we think of language structure as reflecting an accumulated set of changes in our genome, then we might ask something like, “What are the genetic bases of language structure and why were they selected?” However, if cultural evolution can account for language structure, then this question no longer applies. Instead, we face the task of accounting for the origin of the traits that enabled that process of structure-creating cultural evolution to get started in the first place. In light of work on cultural evolution, then, the new question for biological evolution becomes, “How did those precursor traits evolve?” We identify two key precursor traits: (1) the transmission of the communication system through *learning*; and (2) the ability to infer the *communicative intent* associated with a signal or action. We then describe two comparative case studies—the Bengalese finch and the domestic dog—in which parallel traits can be seen emerging following *domestication*. Finally, we turn to the role of domestication in human evolution. We argue that the cultural evolution of language structure has its origin in an earlier process of self-domestication.

## Introduction

The last two decades have seen a resurgence of interest in the evolution of language. In its initial phase (e.g. Pinker and Bloom 1990), much of this work treated language structure as a genetically encoded biological trait. On this view, any structure seen in language simply reflected what was encoded in the genome, as part of a complex adaptation for communication (Pinker and Jackendoff 2005). In recent years, however, a growing body of work has begun to show that many aspects of language structure are the result of *language itself* adapting to constraints imposed by the way it is transmitted (see Kirby 2017; Tamariz and Kirby 2015; Kirby et al. 2014; Dediu et al. 2013 for recent reviews).

This basic finding is now supported by experimental studies and computational modelling into a range of linguistic phenomena. These include the emergence of compositionality (Kirby 2002; Brighton et al. 2005; Kirby et al. 2008); morphosyntactic regularity (Kirby 2002); recursive syntax (Kirby 2002); subjacency (Christiansen et al. 2002); regularisation of variation (Smith and Wonnacott 2010); the emergence of arbitrary signals (Theisen et al. 2010) and discrete phonological units (Oudeyer 2005, 2006); and duality of patterning (Roberts and Galantucci 2012; Verhoef et al. 2014). However, the implications of these findings—what they mean for our wider understanding of the evolution of language—have received relatively little attention. It is these implications, the new questions they raise, and the role of self-domestication in answering those questions that forms our focus here.

We start by considering the kind of questions we might ask of *biological* evolution. Simply put, we see the process uncovered by work on the cultural transmission of language as a kind of ‘informational regularity’, akin to the regularities afforded the evolutionary process by the laws of physics and mathematics (e.g. Kauffman 1993; Goodwin 1994; Stewart 1998). Such regularities have the common feature of providing structure ‘for free’. Given this, it makes little sense—and indeed renders it *unnecessary*—to seek a biological explanation for language structure itself. Instead, the core question for biological evolution should be the origin of the key precursors that make the *cultural* evolution of language possible in the first place.

As a first approximation, we suggest that the emergence of structured language through cultural evolution required two key precursors. The first is that communicative signals need to be *learned from others*, rather than being present from birth. Cultural evolution can only occur if something is learned, transmitted between generations, and changes in response to that transmission. The second is that this learning needs to be guided by a sensitivity to the *communicative intent* of others. Guided, that is, by the ability to recognise when another individual’s movement, gesture or sound was made *in order* to communicate, and what it was *intended* to mean.

In addressing the origin of these precursors, we turn to the resources of comparative biology. In particular, we looked for evolutionary *analogies* (Gould 1976): Instances of similar traits emerging in other, distantly related species, through a process of parallel evolution. We identify two species, each of which exhibits aspects of one of the two precursors. In both cases, these instances of parallel evolution seem to be linked to the *domestication* of the species. The first of these species is the Bengalese finch (e.g. Okanoya 2012), which following domestication has come to rely much more on learning to transmit its song between generations, thus serving as a parallel for the first precursor. The second is the domestic dog (e.g. Hare et al. 2002), which exhibits a particularly acute awareness of when actions or gestures are meant communicatively, thus paralleling the second precursor.

This leads to the question of whether domestication might also explain the emergence of these precursors in humans. The idea that humans are a domesticated species has deep intellectual roots, tracing back at least to classical antiquity (Leach 2007). In the second half of this paper, we present an overview of the concepts, evolutionary processes, and outcomes associated with domestication, together with their applicability to the human case. Central to this discussion is the notion of the *domestic phenotype*: a suite of skeletal, dental, soft tissue, behavioural, and reproductive changes that are common to a wide range of domesticated species. There is now much evidence that humans also exhibit the domestic phenotype.

In summary, we argue that recent work on the cultural evolution of language renders a biological account of language structure unnecessary. Rather than seek a biological account of the emergence of language structure itself, we think the focus should be on the biological underpinnings of the cultural process. A survey of some relevant comparative studies suggest that the conditions typical of *domestication* may play a key role in accounting for how such a cultural process may have managed to get started. Linking this with the growing interest in the role of domestication in human evolution, we suggest that the biological precursors of structure-creating cultural evolution lie in an earlier process of self-domestication.

## The cultural evolution of language

In the late 1990s, a number of researchers began to model the ways in which languages evolve culturally in response to being transmitted through multiple generations of individuals, each of which learned the system through the observation of a subset of other individuals’ signalling behaviour (see Kirby et al. 2014; Smith 2014 for reviews). That the learners only observe a *subset* of the signalling behaviour of the previous generation is key, as this creates a *bottleneck* on the transmission process. In a typical computational simulation of this process, the initial generation of learners were trained on a set of non-compositional, random signal-meaning associations. Those same individuals then went on to produce signals themselves, which then served as the input to the next generation of learners, and so on. This is the core of what is meant by the term ‘iterated learning’. The central question, then, concerned the kinds of language that can survive in a world in which learners are only ever exposed to a subset of a language’s constituent signal-meaning pairs. The core finding of this work was that random languages become compositional over the course of the simulations. Compositional languages survive transmission through the bottleneck, whereas random languages do not, because they are simpler, or more *compressible* (Brighton 2003), and thus easier to learn.

However, this modelling work was vulnerable to two main criticisms. The first was that the apparently emergent structure simply reflected the learning algorithms built into the agents, rather than anything about the transmission process itself. To what extent, then, is there an effect of cultural transmission, over and above that of the influence of the learning biases or algorithms of the learners? The second concerns the applicability of these findings to the real world. To what extent would the results of these simulations be mirrored in work done with human beings?

The first criticism was addressed through the application of Bayesian techniques (Griffiths and Kalish 2007; Kirby et al. 2007). The key contribution of the Bayesian approach lies in the concept of the *prior*, and its ability to make learning biases explicit. This allows us to see what contribution iterated transmission is making, if any, over and above that of individual biases. A crucial finding of this Bayesian work is that there are a range of conditions under which cultural transmission has the effect of *amplifying* learning biases. More specifically, as long as learners possess *some* kind of bias for structure, however weak that bias might be, cultural transmission can serve to amplify the effect of that bias, such that the resulting language is highly structured (Smith and Kirby 2008). Another way to think about this is in terms of the strength of the bias being *masked* (Deacon 2009) by the presence of cultural transmission. As a result, a highly structured language will emerge over the course of repeated transmission, regardless of whether the individual agents have a weak or strong bias for structure. In turn, this sets up an evolutionary process whereby the weakest possible bias in favour of structure is likely to be favoured (Thompson et al. 2016).

The second criticism was addressed through the expansion of iterated learning to experimental studies in the lab (Scott-Phillips and Kirby 2010; Tamariz 2017). The aim of these studies is to replicate the logic of the simulations as closely as possible with real participants. They can be seen, then, as a combination of the kind of artificial language learning experiments seen in psycholinguistics (e.g. Reber 1967) with the diffusion chain paradigm from experimental cultural evolution (e.g. Mesoudi and Whiten 2008). In one of the earlier of these studies, Kirby et al. (2008) trained participants on an artificial language for naming coloured shapes. The initial language consisted of an entirely random set of signal-meaning pairs. Having been trained on half the language—an effective ‘bottleneck’ on transmission—these first participants were then asked to label the full set of shapes, forcing them to recall what they had been trained on and to generalise to the whole set. The output from this first set of participants was then used as the training language for the next set of participants, with this process being repeated for each generation.

Intriguingly, the key insight from this early experimental work was that its results did *not* match those of the simulation studies. As the language was transmitted between generations of participants it did indeed become simpler and easier to learn. However, it did so by becoming a degenerate, or *systematically underspecified,* language, in which a single signal was associated with multiple meanings. Becoming easier to learn through the simple shedding of distinct signals is clearly an adaptation to passing through the bottleneck. However, it is not a realistic account of the emergence of the kind of structured language we see in the world. This discrepancy between the experimental and simulation studies is resolved, however, if participants are placed in an interactive context (Kirby et al. 2015; Winters et al. 2015). The need to be used for *communication* introduces a second pressure into the environment of the language, with the result that languages again became compositional over the course of transmission.

The collective findings of the last two decades work on the cultural evolution of language lead us, then, to identify two key pressures in the environment of the language (Kirby et al. 2015; see also Kemp and Regier 2012). The first is that language must be *learnable*. If a language is too complex or difficult to learn, then it will simply not get passed on with any fidelity. This is a pressure, then, for ever greater *simplicity* (Brighton 2003; Brighton et al. 2005). Against this pressure to simplify, however, lies the fact that language is used to *communicate*. Language must be *expressive* enough to be useful for communication. There is, then, an inevitable trade-off in the form a culturally transmitted language comes to take. The simplest possible language would be one in which a single signal was associated with every meaning, however this would be of little use in communication. Conversely, a language with a unique and unrelated signal for every meaning would permit totally unambiguous communication but be near impossible to learn. Cultural evolution shapes language structure in response to just this trade-off. The process of cultural transmission, with its interplay between the pressure to simplify and the pressure to have communicative utility, generates a compositional language, which is structured to meet both pressures.

This, then, is what we mean when we say that cultural evolution presents as a kind of ‘informational regularity’. The very process of transmission, whether implemented in simulations or in human participants, promotes the structuring of the transmitted system and serves to amplify any biases for structure that may be present in learners. Initially random systems of signals, then, become structured simply by virtue of being culturally transmitted, without any need for a concomitant change in the learners who use the system. It is this sense that structure is provided ‘for free’ to biological evolution. In short, structured systems survive because they are easier to learn. However, as experimental work has shown, the *kind* of structure that results from cultural transmission is not necessarily the kind of structure we see in language. Recall, for example, how under certain circumstances the transmitted system can become systematically underspecified. The compositional structure we see in language is, then, a result of this process of cultural transmission occurring in a context where the learners use the system to communicate. Compositional structure is what results when a pressure for communicative utility is added to a process, cultural transmission, that is itself already structure-creating in nature. This renders an account of language structure rooted in biological evolution unnecessary. Instead, we argue that we should look to biological evolution to provide an account of how this cultural process became possible in the first place.

## The biological precursors of a culturally evolving language

### The learning of new signals

For structure to emerge through cultural evolution, it is necessary that the system be learned from others. However, the communication systems of most species are not transmitted in this way. The pattern across mammals, at least as far as vocal communication is concerned, is that most species have a limited repertoire of signals which are present in their adult form from birth (Seyfarth and Cheney 2010). We should be clear here, however, about what we mean by ‘learning’. When we talk about learning we are specifically talking about *production* learning (Seyfarth and Cheney 2010), where an existing signal is modified or a new signal is acquired. This stands in contrast to *comprehension* learning, which refers to the ability to extract a new meaning or inference from a signal; and *usage* learning, where the usage of a signal is modified based on the current situation or context (Janik and Slater 2000; Seyfarth and Cheney 2010).

There are, of course, examples of production learning found in nature. Among mammals, known vocal learners include some species of whales and dolphins (Reiss and McCowan 1993; Rendell and Whitehead 2001), bats (Boughman 1998), seals (Ralls et al. 1985), and elephants (Poole et al. 2005). We have no doubt that many further examples of mammalian vocal learning will be discovered in the future. Among birds, production learning is found in both parrots (Pepperberg 2010) and hummingbirds (Baptista and Schuchmann 1990). Of course, the most unequivocal evidence of vocal production learning is found in songbirds (Nottebohm and Liu 2010), many species of which require exposure to other singers during development in order to develop species-typical song (Beecher et al. 2010). The importance and widespread nature of learning in songbirds makes them a particularly good ‘natural laboratory’ for the question of how and why a central role for learning might have emerged in relation to language.

While communication through vocal signals is widespread in nature, communication through gesture—that is, through “manual communication without touching another individual or a substrate”—is found almost exclusively in apes and humans (Pollick and de Waal 2007: 8184). The gestural communication of apes is significantly more flexible and less tied to emotional reactions or specific contexts than either their vocal or facial expressions (Pollick and de Waal 2007). The comprehension and usage of ape gestures in the wild is known to shift between contexts (Hobaiter and Byrne 2011), and the emergence of new, non species-typical gestures has been observed in captivity (Leavens et al. 2005). Of course, gesture, although learned, is not the predominant modality of language as we know it today. This, amongst other things, has lead some to suggest that language may have originated in the gestural modality, only later becoming primarily vocal (e.g. Corballis 2002). In contrast, others have suggested that it is not so much that language itself switched modality, but that the same underlying cognitive capabilities that permit the flexibility of learned gesture in apes may have been extended to the vocal domain (e.g. Tomasello 2008).

### Communicative inference: linking signals to meanings

However, vocal production learning is not itself enough. What is required for language is the production learning of new signal-*meaning* associations. There seems little evidence that any of the vocal production learners discussed above are learning new signal-meaning associations, or even that their signals have any semantic content at all (Fitch 2005). Even in one of the clearest examples of vocal production learning, that of the songbirds, there appears to be no evidence that there is any semanticity to the learned song, or that song elements can be rearranged to yield changes in meaning (Berwick et al. 2011). There are, however, some instances of signal-meaning associations being learned in apes. Learning of this kind can be seen in the process of *ontogenetic ritualisation* (Tomasello 1996), in which signal-meaning associations are constructed through repeated interactions. It can also be seen in ape language research (Savage-Rumbaugh et al. 1986, 1998, 2005; Lyn 2007), in the form of learned lexigrams and gestures.

Language is unusual, however, because it is both *learned* and *symbolic* (Deacon 1997). As such, the link between signals and their meanings is neither innately specified nor inherent in the form of the signal (Oliphant 2002). This greatly complicates the task of acquiring new signal-meaning pairs, because it requires not just associative learning between items, but also some way of figuring out what words actually mean. To learn a new signal-meaning pair in a language-like system, then, requires the capacity to *infer* what a communicator *intended* the signal to mean.

In language, the inferential acquisition of new signal-meaning pairs is most clearly exemplified by word learning. Many different processes are likely involved in word learning (Markman 1994; Samuelson and Smith 1998; Saffran 2003; Smith et al. 2011). However, it is the social-pragmatic account (e.g. Tomasello 2000) that has the most to say about the problem of meaning inference. This account is rooted in our awareness of others as intentional agents (Tomasello 1999), and our capacity to engage in joint-attentional activities (Tomasello et al. 2005), against a background of *mutually shared* knowledge, expectations and goals. This background, often referred to as ‘common ground’ (Clark 1996) or in terms of a ‘mutual cognitive environment’ (Sperber and Wilson 1995), creates a situation in which the range of potential referents for a given utterance is drastically reduced. In summary, then, our second precursor is not simply the production learning of new signal-meaning associations, but the ability to acquire these associations through an inference of communicative intent.

However, there is an even more basic form of this precursor, which stands as a requirement for any account of learned symbols to be possible in the first place. This concerns the recognition that an action or behaviour was meant communicatively *at all* (Scott-Phillips et al. 2009). In contrast to inferring the meaning of a particular signal, we might call this a general *sensitivity to communicative intent*: an awareness, that is, that a particular signal or action was made *in order* to communicate. Given that the full suite of capacities underpinning joint-attentional situations and the inference of communicative intent are likely unique to humans, we think it more promising to focus on this more basic form of the precursor.

## The origin of the precursors in domestication

In the following sections, we discuss two comparative studies, which each present as evolutionary analogies of one of the two preconditions for a structure-creating process of cultural evolution. In each of the two examples, the parallel evolution of these key precursor traits occurred in the context of *domestication*. We explore what it is about domestication that likely lead to this outcome.

### The Bengalese finch and the learning of signals

The Bengalese finch is a domesticated strain of the white-rumped munia (Okanoya 2002), a bird native to tropical continental Asia and some of the surrounding islands. For the last 250 years the Bengalese finch has been bred in Japan for its white plumage (Okanoya 2004; Svanberg 2008). Importantly, the Bengalese finch has *not* been bred for its song. Despite this, the song of the Bengalese finch has changed remarkably over the course of its domestication. It is the nature of these changes, together with the reasons why domestication had this kind of effect on its song, that makes this bird significant for those interested in the cultural evolution of language.

The role played by learning in songbirds differs along a number of dimensions (Beecher and Burt 2004; Beecher and Brenowitz 2005; Beecher et al. 2010; Soma 2011). As such, the changes to the Bengalese song brought about through domestication are best appreciated against a backdrop of the similarities between the Bengalese finch and its wild ancestor. Firstly, both the wild and domesticated species are *closed learners* (Okanoya and Yamaguchi 1997; Soma et al. 2006), meaning they can only acquire their species-typical song during a developmental ‘sensitive period’. Secondly, both species require exposure to conspecific song during development (Bao et al. 2003; Peng et al. 2012). Species-typical song will not develop if they are reared in isolation, as it can in some species (e.g. Kroodsma et al. 1997; Leitner et al. 2002). Finally, both the wild and domesticated strains are ‘social learners’, who learn better from conspecifics than from prerecorded ‘tape tutors’ (Eales 1989; Soma 2011).

Both species, then, are vocal learners. Domestication has not turned a non-learner into a vocal learner. What has changed, however, is the role and importance of learning—specifically, learning *from others*—to the transmission of the song between generations. This can be seen in three further dimensions along which the wild and domesticated strains differ. Firstly, the domesticated Bengalese now sings a much more complex and syntactically rich song, with greater levels of unpredictability in the patterns of transition between notes and note groups than is seen in the wild munia (Okanoya 2002, 2012). Secondly, cross-fostering experiments (Takahasi and Okanoya 2010) have shown that Bengalese chicks exhibit much lower copying fidelity in what they learn from tutor birds. Whereas munia chicks copy tutors with a high level of fidelity, Bengalese chicks combine the tutor’s song with their own improvisations and variations. Finally, and most importantly, Bengalese finches are much less constrained in what they are able to learn. Song learning in the white-rumped munia is highly canalized, such that munia chicks are only able to acquire a narrow range of species-specific song. In contrast, Bengalese chicks are much less constrained in what they are able to learn (Takahasi and Okanoya 2010).

Three important points follow from these differences. The first is that the reduction in learning constraints seen in the Bengalese finch means that the specifics of experience during development (e.g. particular tutor used as model) have a much greater influence on the structure of the resulting song. The second is that the reduction in high-fidelity copying combined with the broader range of *what* Bengalese chicks will copy has resulted in a much greater variation in song between different finches than is seen in their munia ancestors. Finally, all three of these differences combined have meant that many Bengalese finches have come to sing songs of much greater complexity than seen in white-rumped munias.

In the wild-living white-rumped munia, we have an example of a stereotypic, highly canalized communication system in which learning plays a minimal role. In its domesticated descendent, the Bengalese finch, song learning is less canalized, the songs themselves are less stereotypic and the influence of traditional transmission on song structure has increased. We see in this example, then, a parallel with the first of the preconditions identified above: an increase in the role of learning and cultural transmission. This change occurred in the context of domestication. Recall, however, that despite this context it cannot be attributed to artificial selection for more complex song. Why, then, might domestication have changed this bird’s song in this way?

One of the major characteristics of domestication is the *buffered* nature of the environment (Zohary et al. 1998; Price 1999, 2002; Deacon 2010), in which organisms are no longer subject to many of the selective pressures typically found in the wild, such as predation, unpredictable variation in food supply, and climatic variation. Deacon (2003, 2009, 2010) has proposed that domestication operated to relax various selection pressures on munia song that had kept it simple and stereotypical in the wild, allowing the song to become more complex under domestication (see also, Ritchie and Kirby 2007). This relaxation of selective pressure, argues Deacon, resulted in a breakdown of the learning biases and other factors that had kept the song simple in the wild and served to restrict the potential role for learning in shaping song characteristics. In turn, this opened up the possibility for learning and other aspects of early experience to influence song structure much more greatly under domestication. It is important to note that this is not the relaxation of selective pressure, per se, such that no selection occurs, but of *specific* pressures that served to restrict the potential contribution of learning and individual experience to the resulting song.

One such pressure concerns the need for accurate species recognition. Kagawa et al. (2012) compared the songs of three wild populations of white-rumped munia on the island of Taiwan. The syntactic complexity of munia song was found to vary in relation to the number of sympatric, closely related species. One of the key functions of song is species recognition, which is important in order to avoid the infertile hybrids that often result from cross-species matings. This is best achieved through the use of simple, stereotypic songs that exhibit little variation. In locations with fewer sympatric close relations, however, the selective pressure on species recognition is relaxed. The greater song complexity found in areas with fewer sympatric species could well be another example of song complexification following a relaxation of selective pressure. Kagawa et al. have, then, identified a key selection pressure that is both relaxed under domestication and found to be related to song complexity in the wild.

The second strand of evidence relates to the differing levels of stress hormones found in white-rumped munia and Bengalese finches. Suzuki et al. (2012) report measurements of fecal corticosterone, a hormone known to be directly involved in the development of the song system (Suzuki et al. 2011). Bengalese finches were found to have lower levels of corticosterone than white-rumped munia, regardless of whether the munia had been wild-caught or captive raised, indicating that it is domestication of the lineage that matters and not simply the conditions in which an individual bird was raised. Indeed, changes in hormonal regulation are known to commonly follow from domestication more generally (Price 2002; Trut et al. 2009). A range of work shows that higher levels of corticosterone negatively affect the development of the song system and can reduce the complexity of the resulting song (Spencer et al. 2003; Buchanan et al. 2004). If this is the case, then the finding that domestication can reduce levels of corticosterone in finches—perhaps through consistently reduced levels of stress in a buffered environment—might well provide a physical mechanism whereby the relaxation of selection following domestication could induce song complexification.

Finally, it is also clear that both female Bengalese finches and female munias have a preference for more complex song (Okanoya 2002). The potential role of sexual selection is somewhat attenuated by the fact that Bengalese breeding has long been under human control, although there is still scope for sexual selection to influence song structure through the higher ‘breeding efficiency’ of bird pairs in which the male sings a more complex song (Okanoya 2004). The precise nature of the interplay between relaxed selection and female preference remains unclear. It may be as simple as the two factors acting to reinforce one another. Of course, we can ask why such female preference for complexity should be satisfied through complexity that is *learned*, rather than, say, the impressive *improvisation* seen in some other species (e.g. Leitner et al. 2002), either of which would fit equally well with the major selective theory of song complexity in birds, the developmental stress theory (Nowicki et al. 1998; Buchanan et al. 2004; Ritchie et al. 2008). One possibility is that the environment of domestication, having already relaxed selection on song simplicity, and thus facilitated a greater role for learning in song transmission, set up just the conditions for a demonstration of fitness through learning rather than through improvisation or other means (Thomas 2013).

### The domestic dog and communicative inference

Starting in the late 1990s a number of studies appeared describing how domestic dogs were particularly adept at using human communicative cues, such as pointing (Hare et al. 1998; Soproni et al. 2001), gaze (Hare et al. 2002), location markers (Agnetta et al. 2000), and even 3D replicas and photographs (Kaminski et al. 2009). Of particular interest was the fact that dogs seemed to outperform chimpanzees and other apes (Hare et al. 2002; Hare and Tomasello 2005; Gómez 2005; Miklósi 2007), and indeed seemed more similar to human children in this respect, although the true capacity of apes in this is a matter of debate (see Mulcahy and Call 2009; Mulcahy and Hedge 2012; Kirchhofer et al. 2012). Furthermore, these abilities are found across a wide range of different breeds (Wobber et al. 2009), including breeds that had been bred as working dogs like retrievers, companion dogs like toy poodles, and even once-domestic but now-feral breeds like the Australian dingo (Smith and Litchfield 2010).

These studies all utilised a variant of the *object choice task* (see Miklósi and Soproni 2006). In this procedure, a piece of food or other desirable item is placed in one of two or more locations. The location of the food is then indicated to the subject through pointing or some other cue, and the subject is then allowed to choose between the locations. The question of interest is whether the subject can use the cue to select the correct location. More specifically, however, what matters is not the ability to respond to the cues per se, but the extent to which a comprehension of the *communicative* nature of the cues is necessary for success on the task. It is quite possible, for example, to be successful with some cues, such as location tapping or sustained, close-in pointing, purely as a result of stimulus enhancement. Other cues, however, such as iconic representations and brief points from more distant locations, are much less salient in this regard. Finally, such comprehension is even more strongly confirmed if responses are modified based on the *ostensive content* of those cues. For example, by responding differently to intentionally given communications than to very similar physical actions produced ‘by accident’.

Studies with wolves and young puppies suggest that this ability in dogs is neither a simple inheritance from the canid line more generally, nor dependent on exposure to humans during development. Miklósi et al. (2003) compared dogs and wolves that had been socialised with humans to a comparable level. They found that dogs significantly outperformed wolves on the object choice task. Virányi et al. (2008) conducted a longitudinal study with sets of hand-reared wolves and dogs. When tested at a young age, the dogs significantly outperformed the wolves despite similar levels of exposure to humans. They then went on to re-test the wolves at regular intervals. The wolves performance steadily increased with each re-testing, such that eventually the best subset of these highly trained wolves reached a comparable level of performance with naive dogs, who had not previously been tested. Echoing Virányi et al’s findings, Riedel et al. (2008) found a similar level of performance across dogs of all ages, including puppies as young as 6 weeks old.

We should note, however, that others have disputed the claims of dogs’ superiority to wolves and the presence of the capacity in young dogs (Udell et al. 2008; Wynne et al. 2008). This has lead to the suggestion of the so-called ‘two-stage hypothesis’, which suggests that dogs’ abilities stem from a combination of an initial exposure to humans during their early socialisation period, followed by extended reinforcement learning over the course of life (Udell et al. 2010). This, then, is something of a domain-general account of how these abilities emerged, in contrast to the more domain-specific account rooted in domestication. However, a range of further evidence suggests that the abilities found in dogs go well beyond what could reasonably be accounted for through a domain-general effect of reinforcement learning.

The most significant of these further findings concerns a number of parallels between dogs and human infants in their response to communicative cues. Firstly, like human infants, but unlike other apes (Hare and Tomasello 2004; Herrmann et al. 2006), dogs appear to show a particular sensitivity to cues in co-operative contexts (Pettersson et al. 2011), rather than in competitive situations. Secondly, dogs, again like human infants, show a particular sensitivity to the ostensive content of signals and cues (Kaminski et al. 2012). Dogs respond differently to intentionally given cues, than to similar actions produced ‘accidentally’, and show sensitivity to a range of ostensive cues, such as establishing eye contact and calling their name. Finally, dogs even exhibit some similar *errors* to those seen in human infants in interpreting communicative cues (Topál et al. 2008, 2009), including the so-called *A*-*not*-*B* error related to object permanence.

We should pause here to note that these abilities have been investigated in a number of species other than dogs, including dolphins (Pack and Herman 2004, 2006, 2007), seals (Shapiro et al. 2003; Scheumann and Call 2004), horses (Proops et al. 2010), and goats (Kaminski et al. 2005). Studies have also been conducted with a number of bird species including parrots (Giret et al. 2009), and numerous kinds of corvids (e.g. Schloegl et al. 2008; Tornick et al. 2011). In many cases the results can be explained in terms of stimulus enhancement, with levels of correct response correlating to the saliency of the cue used. However, in some cases, particularly dolphins and seals, there does indeed seem to be some genuine understanding of the communicative nature of the cues. However, much like with socialised wolves, these more impressive cases typically involve individuals who have had intensive, long-term contact with humans, often participating in research programs, demonstrations or shows for many years. In addition, there have been a number of studies of other domesticated species, including cats, horses and goats (see Miklósi and Soproni 2006; Thomas 2013 for reviews), which have returned somewhat inconclusive results.

Having an evolutionary history of domestication is not, then, a *necessary* condition for the sophisticated utilisation of human communicative cues. However, there may be multiple routes, each comprised of different proportions of phylogenetic and ontogenetic contributions, that can lead to similar phenotypic outcomes (Miklósi and Topál 2011). Broadly speaking, the *ontogenetic* route, taken by dolphins, seals and intensively socialised wolves, consists of long-term exposure to humans. In contrast, the *phylogenetic* route, seemingly taken by the dog over the course of domestication, means it requires little or no exposure to humans for comparable capacities to become manifest (Miklósi and Topál 2011). We are left, then, with much the same question as followed from the case of the Bengalese finch: what is it about the process of domestication that caused this change in dogs? Fortunately, however, there is a long-running experiment, expressly designed to investigate the domestication of the dog.

The farm fox experiment (Belyaev 1979; Trut 1999; Trut et al 2009) was started in 1959 by the Russian geneticist Dmitry K. Belyaev. The experiment took the Siberian silver fox—a regional variant of the more familiar red fox—as its model animal, and began a selective breeding program, still running today, to recreate the domestication of the dog, and to investigate the origins of the physical and behavioural characteristics typical of domesticated species. At the core of the experiment is the breeding of three lines of foxes, tame, aggressive, and a control group. For reasons of clarity and space we will focus on the tame-line foxes.

Selection in the tame-line foxes was solely based on their temperament, as assessed through their reactions to humans (Kukekova et al. 2006, 2008, 2012). Foxes were then classified into groups based on their overall aggressive behaviour, with the tamest, least aggressive foxes known as the ‘domesticated elite’. The selective pressure applied to the tame line of foxes was very strong, with only the top 10% of most tame individuals being allowed to breed (Trut et al. 2009). Unsurprisingly, this rapidly increased the percentage of foxes classified as ‘domesticated elite’, from 1 to 2% at the beginning of the experiment to almost the entire population after fifty or so generations (Trut et al 2009).

What is perhaps more surprising, however, was the range of other changes that also occurred in the tame line of foxes, as listed in Table 1 (after Trut 1999; Kukekova et al. 2006; Trut et al. 2009; Bidau 2009).

**Table 1:** Correlated phenotypic changes following selection on temperament

Earlier response to sound	Piebald coat
Eyes opened earlier	Brown mottling on coat
Delayed development of fear response	Floppy ears
Extended socialisation period	Shortened tails
Play extended into adulthood	Curly tails
Earlier sexual maturity	Smaller cranial height and width
Breakdown of strict mating seasons	Decrease in sexual dimorphism

The most striking thing about this list is how many of these changes are typically found in domesticated species (Price 1999), forming part of the *domestic phenotype*. One remarkable finding of the farm fox experiment, then, is that many of these typical outcomes of domestication can be produced simply as a by-product of selection against aggression. For present purposes, however, the most important change that occurred in the tame line of foxes was that, like domestic dogs, they also came to exhibit a sensitivity to communicative intent.

Hare et al. (2005) conducted an object-choice task, similar to those described above, comparing the abilities of dog pups, tame-line domesticated fox kits and control fox kits. The three groups were tested on their ability to use a point-and-gaze cue to select the correct location of some hidden food. The two major findings were that tame-line fox kits performed as well as dog puppies, and that the tame-line kits outperformed kits of the control population. There was also no evidence of learning during the experiment, as the tame-line kits performed as well in the initial trials as in later ones.

Temperament is the only criteria on which these foxes were selected. The fact that the sensitivity to communicative intent has emerged in the tame fox line lends support, therefore, to the *emotional reactivity hypothesis* (Hare et al. 2005; Hare and Tomasello 2005; Melis et al. 2006). This is the view that cognitive changes, particularly those involving co-operative behaviour, may not always requires direct selection, but can appear as a by-product of selection acting on systems of emotion or aggression that had previously prevented the use of preexisting skills in these kinds of co-operative contexts. This speaks directly to the question of why and how domestication might have resulted in this ability emerging in dogs. The answer arising from the farm-fox experiment is that such capacities are likely to have emerged as a by-product of selection targeting defensive and aggressive behaviours.

## Bridging the ‘gap’ to humans

In the Bengalese finch, relaxed selection, changes in the regulation of stress hormones, and female preferences have combined to expand the role played by learning. This provides a parallel to the first of our precursor traits, regarding the importance of *learning* in the transmission of a communication system. Recall that learning plays little role in the transmission of most species’ communication systems. The Bengalese finch provides us with a documented case study of how learning might take on a greater role. In the domestic dog, selection on temperament has enabled the emergence of a particularly acute sensitivity to communicative cues. This serves as a parallel to our second precursor trait, that the *kind* of learning required for a system like language is one that is fundamentally rooted in communicative inference. Of course, neither the Bengalese finch nor the domestic dog provide a full analog to their respective traits in humans. It is, after all, no surprise that the full depth and complexity of language learning and human social cognition would not be present in other species. However, in both instances we see the parallel evolution of the core elements of the two precursor traits which we identify as underpinning the cultural evolution of language structure. We think the fact that *both* these instances of parallel evolution occurred in the context of domestication provides an important clue as to how these key precursor traits might have evolved in humans.

However, we also acknowledge that there remains a significant explanatory “gap” between humans and language on the one hand, and the two case studies of domestication on the other. If we were to be critical of our argument so far, we might put it somewhat like this. What we have is two “pieces” that appear to fit together: the preconditions required for a structure-creating process of cultural transmission, and the two case studies of domestication in which parallels to those preconditions can be seen emerging. What remains to be demonstrated is whether, and even *how*, these two pieces might be part of the same “puzzle”. Several questions naturally arise here. What is ‘domestication’? What has domestication got to do with human evolution? How could domestication-like changes have occurred in humans?

### What is domestication and why is it relevant to humans?

Why we should even *consider* the possibility of domestication having played a role in human evolution? After all, does not domestication require that there be a domesticat*or*—an outside agency selectively breeding the species? In this section we contrast two conceptions of domestication (see Thomas 2013). (1) The *conditions* view, in which domestication is characterised in terms of being under the control of another species. (2) The *outcomes* view, in which domestication is characterised by the typical traits that are shared by many domesticated species, known as the *domestic phenotype*.

#### The *conditions* view of domestication

The view of domestication held by many people is probably well captured by the following quote:

[a domestic animal is] bred in captivity for the *purposes* of subsistence or profit, in a human community that *controls* its breeding, its organisation of territory and its food supply.

(Clutton-Brock 1992: 41, our emphasis)

As the emphasis makes clear, this view focuses on domestication as the human ‘mastery’ of nature, through the control of other species, by humans, for our own conscious purposes. To an extent, of course, this description of domestication is accurate. However, it also brings with it a number of problems.

For one, while it is an accurate description of the current-day living conditions of many domesticated species, it is an entirely insufficient account of *how* those species came to take on their present-day characteristics. This is because many aspects of the domestic phenotype can be traced not to selective breeding but to continuing natural selection under domestication (Price and King 1968; Price 1999). The environment of domestication is characterised by reduced living space, increased predictability of food and water supply, dietary changes, an altered social structure, and greater availability of shelter from the elements, resulting in profound changes to an organism’s microclimate (Price and King 1968; Carlstead 1996; Price 1999). Against this backdrop, major evolutionary changes should be expected even in the total absence any artificial selection. A range of domestication-typical changes in mammals, birds, and fish have been associated to some degree with natural selection under domestication. These include reductions in body size (Tchernov and Horwitz 1991); reductions in cranial and skeletal robusticity (Zohary et al. 1998; Houde et al. 2010); reduced sexual dimorphism (Polák and Frynta 2009, 2010); reduced brain size (Kruska 2005); the breakdown of seasonal breeding patterns (Price 1999; Tchernov and Horwitz 1991); and changes in temperament, environmental reactivity, and predator vigilance (Håkansson and Jensen 2008; Campler et al. 2009).

In addition, the conditions view of domestication has the tendency to make us view it as a unitary process. Historically, however, there have been a number of ‘pathways’ to domestication (Zeder 2012). These are as varied as the *prey pathway* where a previously hunted animal comes under direct human control, as was the case with sheep goats, and cattle; and the *commensal pathway*, where the process of domestication is initiated *by the domesticated species itself* in coming to live among humans, as was the case for dogs (Morey 1994). Finally, the systematic application of selective breeding is a recent development in the long history of domestication (Leach 2007), which is measured in tens of millennia. All of this is not to say that artificial selection and selective breeding are unimportant. Rather, the point is that the domestic phenotype cannot be *reduced* to the product of selective breeding. It is the outcome of a range of evolutionary processes taking place against a particular environmental backdrop, much of which has long been shared by humans themselves.

#### The *outcomes* view of domestication

In contrast to the view described above, it is also possible to view domestication in terms of its typical evolutionary outcomes. It has long been known that many phenotypic similarities can be seen across a wide range of domesticated species (Darwin 1868; Price and King 1968; Price 2002). This suite of phenotypic changes has come to be known as the *domestic phenotype*. The following tables list some of its main characteristics, and should be read in terms of how domesticated species typically differ from their wild equivalents (Tables 2, 3). The tables are based on overviews by Leach (2003), Price (1984, 1999, 2002), Clutton-Brock (1999) and Trut et al. (2009).

**Table 2:** Hard tissue changes in the domestic phenotype

Skeletal	Cranial
*Hard tissue changes*	
Reduction in body size	Reduced cranial robusticity
Decreased skeletal robusticity	Reduced brain size
Reduced sexual dimorphism	Shortened facial region
	Greater diversity size/shape horns
	Reduced tooth size
	Tooth crowding/malocclusion
	Juvenile shape retained in adulthood

**Table 3:** Soft tissue and behavioural changes in the domestic phenotype

Physiological	Sexual and life history	Environmental responsiveness
*Soft tissue and behaviour changes*		
Increased variation in coat colour	Earlier sexual maturation	Reduced motor activity
Increased variation in hair structure	Extended breeding seasons	Reduction in information acquisition systems
Enhanced physiological performance	Increased sexual stimulation	Reduced intra-specific aggression
	Greater/more varied litter sizes	Increased docility
	More multiple births	
	Juvenile behaviours retained into adulthood	

This view of domestication is, of course, not incompatible with the conditions view; however, a focus on the evolutionary outcomes of domestication has a number of advantages as as general ‘organising framework’ for thinking about domestication in general, and about the possibility of human self-domestication in particular. Firstly, by focusing on the outcomes of domestication it remains agnostic about the processes and pathways that lead to those outcomes. Secondly, it provides an objective set of criteria for assessing whether a given species is indeed ‘domesticated’. Indeed, the domestic phenotype is used by archaeologists as diagnostic of domestication having occurred in the past (Zeder et al. 2006). Finally, it allows us to re-frame the question of human self-domestication in very concrete terms, and away from potentially unhelpful metaphorical formulations. Humans can be considered domesticated to the extent that they: (1) share in the domestic phenotype; and (2) that those phenotypic similarities have arisen in response to similar evolutionary circumstances and selective pressures, and are underpinned by similar biological mechanisms.

### The domestic phenotype in humans

The idea that humans are a ‘self-domesticated’ species has deep intellectual roots, tracing back at least to classical antiquity (Leach 2007). Over the centuries this view has picked up a number of unpleasant political associations (Brüne 2007). However, from a scientific perspective the main driver of the idea has been the observation that humans, too, share many aspects of the domestic phenotype. This observation can be seen in the writings of Charles Darwin (1871), the anthropologist Franz Boas (1938), and any number of more recent scholars who have compared aspects of human evolution to the outcomes of domestication (e.g. Ashley Montagu 1955; Gould 1977; Leach 2003, 2007; Hare and Tomasello 2005; Deacon 2009, 2010; Bednarik 2012). Unlike most domesticated species, modern humans have no living ‘wild’ ancestor against which their phenotypic traits can be compared. As such, most of these observations compare the modern human phenotype with trends over the course of human evolution, as seen in the fossilised remains of human ancestors, or, where this is not possible, with their closest living relatives, the great apes.

Modern humans have shown a marked decrease in skeletal and cranial robusticity over the last 100,000 years (Ruff et al. 1993; Lahr and Wright 1996; Leach 2003; Bednarik 2012). They have also seen a significant reduction in teeth size (Brace et al. 1987), and in the occurrence of tooth-crowding and malocclusion (Larsson et al. 2005; Leach 2003). Compared both to extant great apes and to ancestral human species, modern humans exhibit a significant retention of juvenile characteristics into adulthood (Gould 1977; Shea 1989; Zollikofer and Ponce de León 2010). In recent years the evidence of neoteny in modern humans has expanded to include aspects of gene expression in the brain (Somel et al. 2009, 2012; Liu et al. 2012), and the timing of *synaptogensis* (Bufill et al. 2011). Modern humans also exhibit very low levels of sexual dimorphism compared both to other apes (Plavcan 2012) and ancestral species of hominids (Harmon 2006; Gordon et al. 2008; Kimbel and Delezene 2009). Unlike other great ape species, human females do not have distinct ‘breeding seasons’, and thus exhibit a form of ‘extended sexuality’ (Rodríguez-Gironés and Enquist 2001), notwithstanding differences in fertility and preferences across the oestrus cycle (Gangestad and Thornhill 2008). There are also early signs that humans may differ in temperament to the other great apes, in ways similar to domesticated species (Herrmann et al. 2011). Finally, it seems that this suite of changes is linked, representing the systemic impact of an underlying mechanism (Trut et al. 2009; Bidau 2009; Wilkins et al. 2014). Evidence is now emerging for a similar links between features such as cranial robusticity, temperament, and neoteny in humans (e.g. Cieri et al. 2014).

Documenting the full range of these parallels, together with the nuances of the arguments over the validity of each one, is beyond the scope of what we can manage here, and the interested reader is referred to the references cited above, particularly Leach (2003, 2007), together with the much fuller version of this discussion in Thomas (2013). We should also mention the one aspect of the domestic phenotype which humans certainly do not parallel: a reduction in brain size. Rather than see brain size reducing, the direction of human evolution has been towards an *increase* in brain size (Rightmire 2004), with any trends in the opposite direction linked to a concomitant reduction in body size (Ruff et al. 1993). It may be that this is one trait where the difference between domestication and *self*-domestication is actually important, with humans, as both constructors and inhabitants of their environment, not subject to the same reduction in stimulation and opportunities for sensory exploration (Price 2002) experienced by other species living in that environment (Leach 2003).

## How might humans have come to share in the domestic phenotype?

The fact that humans exhibit many aspects of the domestic phenotype is the primary reason why the idea of human self-domestication should be taken seriously. However, this still leaves open the question of *how* these parallels might have occurred. In this last section we provide a brief tour of several areas of research aiming to address this question. We first consider aspects of the selective environment that might account for these parallels, focusing on the role of *adaptation to the human*-*made environment* and *selection against aggression*. We then review some evidence regarding the biological mechanisms underpinning the domestic phenotype.

### The selective environment of domestication

As discussed above, many aspects of the domestic phenotype are linked to ongoing natural selection in the human-made environment, with the dramatic changes in living space, food availability and type, microclimate, elemental shelter, etc. that such an environment introduces. What is less commonly recognised, however, is that it is *humans themselves*, as nature’s quintessential niche constructors (Odling-Smee et al. 2003), who have likely been affected most by this environment, given that they have lived in it longest of all. Indeed, as Leach (2003) notes, many of the explanations for the domestication-typical changes seen in human beings, particularly in the last 50,000 years or so, point to aspects of this human-made environment, such as increasing sedentism and associated reductions in activity (Ruff et al. 1993), changes in climate and microclimate (Pearson 2000), and dietary shifts (Cohen and Armelagos 1984; Lieberman 1996). Similar changes in response to the human-made environment have also been observed in commensals—species who live *with* us but are not controlled *by* us—such as the house mouse (Tchernov 1984), and in the ‘inadvertent domestication’ observed in captive breeding programs for endangered species (O’Regan and Kitchener 2005). Once it is recognised that many aspects of the domestic phenotype are associated with the adaptation to a human-made environment, the idea that humans might share those ‘domesticated’ traits comes to be much easier to understand.

The second key factor is the role played by selection against aggression. One of the most important contributions of the farm fox experiment to our understanding of domestication is the extent to which the domestic phenotype can emerge through a ‘correlated cascade’ of changes following selection on temperament. One question that arises from this is whether there are any examples of a similar set of changes following natural selection in the wild. Hare et al. (2012) present a range of evidence suggesting that the bonobo is just such a case. Bonobos differ from chimpanzees along a number of physical (Cramer 1977; Zihlman and Cramer 1978; Pilbrow 2006), behavioural and temperamental (Hare et al. 2007; Hare and Kwetuenda 2010) dimensions that closely parallel the differences between wild and domesticated species. Hare et al. (2012) argue that these differences are ultimately rooted in aspects of the bonobo’s feeding ecology, which have had profound implications for the structuring of bonobo society, especially the favouring of greater co-operation and reduced levels of aggression. The bonobo, then, may be a wild analogue to the proof-of-concept findings of the farm-fox experiment. Furthermore, it may also serve as something of a template for how selection against aggression could be linked to the domestic phenotype in humans. In particular, a growing body of work is now citing changes in human feeding ecology, primarily our shifting to a cooked and processed diet (Wrangham et al. 1999; Wrangham and Conklin-Brittain 2003; Wrangham 2009), as a potential source of similar selective pressure in favour of co-operation and reduced aggression. This possibility is clearly more speculative than the impact of the human-made environment. However, in the farm-fox experiment we have confirmation that this kind of selective regime *can* result in the domestic phenotype, and in the bonobo we have a close relative, for which there is good evidence that a similar process, this time of natural selection, has had a similar phenotypic outcome.

### The physical mechanisms underpinning domestication

We now turn to the mechanisms underpinning the domestic phenotype. However, before our brief review of work in this area, it is worth saying something about the *criteria* such a mechanism has to meet. The domestic phenotype has two key features: the range of species in which it has been observed, and the seemingly disparate set of traits of which it is comprised. To account for the domestic phenotype, therefore, any proposed mechanism must be both highly conserved across species *and* capable of explaining how such an apparently unconnected set of traits so frequently occur together. Follow-up studies on the mechanisms at work in the farm fox experiment has identified changes in the domesticated foxes’ neuroendocrine system as being of fundamental importance (Trut et al. 2009). In particular, a reduction in the production of glucocorticoids and other stress hormones, together with changes in the levels of neurotransmitters such as serotonin. The importance of the role played by the neuroendocrine system is also supported by work with the Bengalese finch (Suzuki et al. 2011, 2012), bonobos (Surbeck et al. 2012a, b), and domesticated species more broadly (Price 2002).

This neuroendocrinal mechanism meets one of the two criteria: the systems involved are highly conserved across species (Bidau 2009). However, as others have noted (e.g. Wilkins et al. 2014), it does less well against the second criteria: it is unclear how such neuroendocrinal changes account for the diverse range of traits that comprise the domestic phenotype. Wilkins et al. (2014) argue that this diverse set of traits, including the neuroendocrinal changes, are linked by shifts in the development, migration, and interaction of Neural Crest Cells (NCC), a vertebrate-specific class of the developmentally important stem cells. They review a wide range of clinical and experimental work which shows similarities between aspects of the domestic phenotype and the effects of genetic disorders, so-called *neurocristopathies*, that affect the generation and function of NCC. Importantly, Wilkins et al. distinguish between NCC as the shared *developmental* basis linking the various traits of the domestic phenotype and their emergence over ontogeny, and the polygenic nature of the underlying *genetic* explanation. This allows them to present a unified account of the diverse traits of the domestic phenotype without needing to talk in overly simplistic terms of ‘domestication genes’.

More recently it has been suggested that changes in the development and regulation of NCC are linked not just to the domestic phenotype but also to the structural ‘language readiness’ of the human brain (Benítez-Burraco et al. 2016). In particular, Benítez-Burraco et al. argue for a link between changes to the NCC and the development of the human-typical ‘globular’ brain shape. This builds on previous work in which they have argued that the distinctive globular shape of the human brain is linked to key features of its modern-day patterns of neural connectivity (Boeckx and Benítez-Burraco 2014a, b), which in turn facilitate what they term ‘cross-modular’ thinking. In linguistic terms, this is exemplified by something like the syntax-semantics interface. In more general terms, it relates to the capacity to make links across cognitive domains, something which may be core to the uniqueness of modern human cognition (e.g. Mithen 1996; Hauser 2009). This work is obviously in its very early stages, but is particularly intriguing regarding the parallels it offers with our work on the mechanism and necessary biological foundations for the cultural evolution of language.

### Why has domestication had this effect on *humans* and not other species?

If domestication set the stage for the cultural evolution of language, it is quite reasonable to ask why *language* itself is not part of the domestic phenotype. Why is something ‘language like’ not seen in other domesticated species? Focusing just on our two central case studies, why do we only see one of the two precursor traits in each instance, and yet humans exhibit both together? These are difficult questions, for which we do not pretend to have definitive answers. However, we think the following points are worth taking into consideration.

Much as we have focused on their similarities, there is also a need to acknowledge the *differences* between domesticated species. One important way in which they differ is the ‘pathway’ they take towards domestication. Some species, like cattle and sheep, are former prey animals that have been slowly corralled into our system of agriculture. Others, like dogs, began the process as freely associating commensals. In the human case, the process of domestication was one of *self*-domestication. We have already discussed the potential consequence of this fact in terms of human brain size increasing, rather than the typical domesticated pattern of reducing brain size. We are highly sceptical of any attempt to draw direct links between brain size and particular capabilities. However, it is at least plausible that this increase in brain size is one contributing factor to the emergence of language (see MacWhinney 2005).

Another way in which domesticated species differ is in terms of their evolutionary history prior to domestication. The evolutionary histories of many lineages have rendered them unamenable to domestication at all (Diamond 1997). Furthermore, if there is a key commonality between the Bengalese finch and the domestic dog, it is that domestication has acted to unleash ‘potentials’ that were already there in the ancestral population. The white-rumped munia is a vocal learner, but freed from selection to keep songs simple and canalized, the role of vocal learning expanded. The grey wolf exhibits sophisticated social cognition, and can reach dog-like levels of performance given extensive contact with humans and repeated exposure to the object-choice task, but does not seem to learn new signals. In the human case, might the combination of primate social cognition with, at least in the gestural realm, the capacity of primates to learn new signals, explain why both precursors emerged together?

We recognise that these brief thoughts can barely begin to address this question. However, we think there are ways in which experimental work could be done in this area. For example, as noted above, many of the more particular impacts of domestication take the form of unleashed potentials. More precisely, potentials that have thus-far been limited by aspects of temperament. It should be possible to identify what these might be in particular instances. For example, Melis et al. (2006) found that chimpanzees who were seemingly unable to solve a co-operative dyadic task could do so if dyad-pairing was manipulated such that individuals with mutually high tolerance were paired together. The poor performance of chimpanzees, relative to dogs, on tasks of co-operative communication stands, then, as something that might be remedied through a change in chimpanzee temperament.

### Summary

We have not attempted to present a comprehensive overview of human self-domestication. Instead, we have focused on the more modest task of trying to close the perceived ‘gap’ between the two sets of data that form the core of this paper: the preconditions required for a structure-creating process of cultural transmission, and the two case studies of domestication in which parallels to those preconditions can be seen emerging. We hope we have helped close it somewhat in the following three ways. First, in focusing on the domestic phenotype we aim to root the idea of humans as domesticates in a concrete, coherent, and falsifiable framework. The focus on a particular set of traits, the domestic phenotype, and the evolutionary explanations for those traits allows us to move beyond metaphorical formulations of what it means to be ‘self-domesticated’. Second, we have identified two evolutionary circumstances—adaptation to the human-made environment and selection on temperament—that are known to contribute to the emergence of the domestic phenotype in other species. The first of these has definitely been a major factor in human evolution; the role of the second, while more speculative, is supported by a range of comparative and archaeological evidence. Finally, we have reviewed a range of work on the biological mechanisms underpinning domestication. These mechanisms are highly conserved—and thus present in a wide range of species, including humans—and can account for the diverse traits of the domestic phenotype. We also touched on some recent work suggestive of a link between the mechanisms mediating the domestic phenotype and language itself.

## Conclusion

There is now a wealth of evidence showing how language structure emerges through a process of cultural evolution. However, the wider implications of this work have received insufficient attention. In particular, our growing knowledge of the role played by cultural evolution has significant implications for what we should expect biological evolution to account for in the emergence of language. Rather than accounting for language structure itself, the key task for biological evolution lies in accounting for the foundational traits that make a process of structure-creating cultural evolution possible. We identified two key traits: the central role of *learning* in the transmission of the communication system; and the ability to recognise the *communicative intent* of a signal or action.

In the Bengalese finch and the domestic dog we have two comparative case studies, each of which show one of these traits emerging in the context of domestication. Two key features of the domestication process stand out as particularly important in accounting for these instances of parallel evolution. The first concerns the *relaxation* of various selection pressures that had been important in the wild. The second concerns the systemic impact of selection acting on the biological systems underpinning temperament and aggression.

Humans share many of the hallmarks of a domesticated species. Much of human evolution has taken place in just the kind of human-made, selection-buffering environment shared by domesticated species. There is also good evidence that humans may have undergone a similar kind of selection on temperament. Given these parallels, we think the two case studies speak directly to the origin of these precursor traits in humans. The cultural evolution of language structure is rooted in an earlier process of self-domestication.
